# The contribution of monocytes/macrophages to HTLV-1 infection and persistence

**DOI:** 10.1186/1742-4690-11-S1-P123

**Published:** 2014-01-07

**Authors:** Maria Fernanda de Castro-Amarante, Cynthia Pise-Masison, Katherine McKinnon, Raya Massoud, Steven Jacobson, Genoveffa Franchini

**Affiliations:** 1 Animal Models and Retroviral Vaccines Section, National Cancer Institute, Bethesda, MD, USA; 2 Viral Immunology Section, Neuroimmunology Branch, National Institute of Neurological Disorders and Stroke, Bethesda, MD, USA

## 

Peripheral blood monocytes can be classified into three main subsets: CD14^++^CD16^–^ (classical), CD14^+^CD16^++^ (non-classical), and CD14^++^CD16^+^ (intermediate) which exert important roles in innate and adaptive immunity. Here we investigated whether the different monocyte subsets are potential HTLV-1reserviors that contribute to viral infection and persistence. PBMCs from 17 HTLV-1 infected patients (IP) and 11 normal donors (ND) were phenotypically analyzed by flow cytometry. Classical monocyte frequency was lower in HTLV-1-IPs compared to NDs (p=0.048). Moreover, we found a positive correlation between PVL and intermediate monocyte frequency (r=0.6735, p=0.0042). Focusing on the presence of HTLV-1 provirus DNA in the different monocyte subsets, cell populations were isolated from PBMCs of 16 HTLV-1-IP. When we analyzed by nested PCR genomic DNA isolated from sorted CD4+, CD8+ CD14^++^CD16^–^, CD14^+^CD16^++^, and CD14^++^CD16^+^, we found that HTLV-1 patients with high PVL, all monocyte subsets as well as CD4+ and CD8+ cells were positive for HTLV-1. In contrast, the intermediate monocytes were negative or very weakly positive for HTLV-1 in patients with low PVL. To test whether natural STLV-1 infection recapitulates what we find in HTLV-1-IPs, we analyzed the monocyte subsets distribution in 8 STLV-1 infected Rhesus macaques and 16 naïve animals. Consistent with human infection, the frequency of intermediate monocytes was higher in infected macaques compared to naïve animals (p=0.0001) with a positive correlation between PVL and intermediate monocytes frequency (r=0.6530, p=0.03). In conclusion, our results suggest that monocytes play an important role in viral dissemination and persistence and are potential viral reservoirs.

